# Editorial: Caring for Those Who Are Neglected and Forgotten: Psychiatry in Prison Environments

**DOI:** 10.3389/fpsyt.2020.00126

**Published:** 2020-03-31

**Authors:** Annette Opitz-Welke, Norbert Konrad, Birgit Völlm

**Affiliations:** ^1^Institute for Forensic Psychiatry, Charité Medical University of Berlin, Berlin, Germany; ^2^Klinik und Poliklinik für Forensische Psychiatrie, Universitätsmedizin Rostock, Rostock, Germany

**Keywords:** mental health, prison, risk, suicide, violence, psychopathy

Prison psychiatry is an area of the psychiatric care system, known only to the few mental health professionals who are working behind bars. As a consequence, the majority may feel unsecure when his or her patient had or has to serve a prison sentence. The aim of this Research Topic is to give an impression of the special challenges of psychiatric work inside correctional institutions.

In general, psychiatric care in prison environments has to deal with a disproportionate burden of psychiatric disease, social marginalization, and substance abuse disorders as well as an elevated prevalence of specific somatic disorders such as infectious diseases. Regarding diagnostic approaches according to the findings of Schildbach, severe mental disorders of prisoners cannot be efficiently detected with the computer aided short screening questionnaire of the DIA-X with a processing time of only a few minutes. Therefore, authors recommended use of the long version of DIA- X interview to open the possibility to identify mental health needs and establish appropriate treatment for those who need it (Schildbach and Schildbach). Although the high prevalence of psychotic disorder, depression, and substance abuse related disorders in prisoners is well-known, evidence about the prevalence of Attention Deficit Hyperactivity disorder in prison environments is scarce. Baggio et al. conducted a systematic review and meta-analysis which pooled 102 original studies including 69, 997 participants. According to their findings the rate of adult ADHD of people living in detention was 26.2%, a 5-fold increase compared to the general population (Baggio et al.).

Prisoners with severe mental disorders have been considered as a vulnerable group of inmates who are especially prone to make false confessions during court procedures. In line with these findings Volbert et al. studied a group of 153 patients of forensic hospitals and found evidence that 25% of the all participants have made at least one false confession.

Violent behavior is a major problem in correctional settings and many measures of restrictive prison routine aim to prevent violence. There is evidence that individuals who suffer from severe mental disorders are in general at higher risk for acting violently. In a retrospective case control study Seidel et al. examined a group of 210 individuals who had acted violently during a stay on a psychiatric ward of a prison hospital and compared them to a group who had never showed violent behavior. A diagnosis of schizophrenia, non-German nationality, no use of an interpreter, having no children and not being previously sentenced were associated with behaving violently during hospital stay (Seidel et al.). Regarding the role of citizenship Neumann et al. examined foreign national and German patients at a prison and compared them to foreign national and German patients at a forensic hospital. Differences in diagnosis were only found in the forensic hospital where foreign nationals were more often diagnosed with schizophrenia. Regarding self-harm foreign nationals were more likely to commit self-harm than Germans during their stay at the prison hospital psychiatric ward (Neumann et al.). Because of the high prevalence of violent behavior in prison environments, there is an urgent need for screening instruments which are capable to detect individuals at risk of acting violently. Negatsch et al. compared a patient group at a psychiatric ward in a prison hospital who had acted violently at least once to a group who never showed any violent behavior. The risk assessment tool OxMIV succeeded in predicting violent behavior in patients suffering from schizophrenia or bipolar disorder, and may thus be useful in detecting individuals at risk for behaving violent in correctional settings (Negatsch et al.).

Although violence is an important issue for prison administrations, prisoners in general are also a high-risk population for self-harming behavior and suicide. The fact that the suicide rate in prisoners compared with that in the general population is significantly higher can also be considered to be an expression of increased mental vulnerability of prisoners. Voulgaris et al. compared suicide rates in forensic psychiatric hospitals and prisons and found no significant difference. To our knowledge is this the first study that presents data for suicide rates within German forensic hospital care (Voulgaris et al.).

While during the last century prison was mostly a matter of young men, the numbers of elderly prisoners are constantly rising. Elderly prisoners are considered a vulnerable prison-subpopulation due to their lack of physical strength and because of their generally poor health. Analyzing data from a survey that included all German prison suicides from 2000 to 2013 Opitz-Welke et al. found higher suicide rates in elderly prisoners in comparison to the general population of the same age. When compared to younger suicide victims elderly suicide victims were more likely to be female, of German nationality, had remand status or were serving a life sentence (Opitz-Welke et al.). As suicide prevention is a major subject of prison mental health care the use of suicide screening instruments is widely recommended. Dezsö et al. described and examined the implementation of a suicide risk screening instruments (SIRAS) in a remand prison and found evidence for a shift in specific interventions toward toward the high-risk group.

Often psychiatry in prison environments provides treatment for those who do not have access to community-based healthcare systems or for those suffering from stigmatizing disorders like sexual deviations. Since sexual fantasies are a key factor in sex offender treatment programs Bartels et al. tested the validity of the Wilson-Sex-Fantasy-Questionnaire for the use with men who have sexually offended against children. The results of this study suggest that the two child-related items of the Wilson Sex Fantasy Questionnaire were more useful than just assessing broad fantasy themes (Bartels et al.).

Providing a therapeutic atmosphere inside correctional facilities is sometimes difficult. In an analysis of the impact of social climate on the treatment outcome in correctional treatment units, Sauter et al. showed that measures to improve team climate and working relationship have an impact on inmates as well. Regarding ratings of inmates' prison behavior by prison officers Hausam et al. studied a group of 272 sexual and violent offenders and found that prison officers behavioral ratings can improve risk assessment.

There is sound evidence that the prevalence of antisocial personality disorder (ASPD) and psychopathy in correctional settings is very high and that individuals with ASPD are difficult to treat and pose a high economic burden on society. Brunner et al. did an analysis of a sample of 205 incarcerated male adults who had been admitted to social-therapeutic facilities in German prisons. Treatment dropouts showed a significantly higher risk of having psychopathy personality traits (Brunner et al.). Lehmann et al. examined a group of 215 violent male offenders with the Psychopathy checklist (PCL-R) factor scores. Results indicated four latent classes with differences of recidivism risk, criminogenic needs and general, violent, and sexual reoffending. According to the authors findings may have implications for the issue of treatment amenability (Lehmann et al.). Looking for new therapeutic approaches for individuals suffering from ASPD there is some evidence that treatment with oxytocin may have a benefit in treating individuals with psychopathy. In a systematic review of the literature exploring the potential use of oxytocin in managing ASPD Gedeon et al. revealed that there were diversified effects with oxytocin showing some benefits and some non-desirable effects in managing ASDP and the symptoms of ASPD. To their opinion further high-quality large sample studies are required before the use of oxytocin may become a treatment option for individuals suffering of ASPD.

After a prison stay good transitional preparation preceding release seems to reduce the risk of poor mental health outcome but is hard to achieve. In a naturalistic prospective observational cohort study Smith et al. could provide evidence for the effectiveness of a Pre-Release Planning Programme of sentenced mentally disordered offenders. Schildbach examined four samples of 100 compensation prisoners each from 1999 till 2017. The majority were homeless, single, and unemployed, exhibited a high degree of substance abuse and showed an extraordinarily high prevalence of mental disorders. Because the average stay of compensation prisoners is short, social rehabilitation after imprisonment is lacking. Schildbach pointed out that compensation imprisonment leads to inappropriate transinstitutionalization and further criminalization of poor or mentally ill people and recommends from a criminalistic perspective community service instead of compensation imprisonment (Schildbach and Schildbach). For improving transition management from prison to community healthcare knowledge about cost effectiveness of mental health in correctional facilities seems crucial. Sridhar et al. reviewed prison healthcare expenditure internationally and found a lack of comparability. They developed a set of consistent and transparent guidelines for consistent and transparent reporting of healthcare costs (Sridhar et al.). The challenges of the treatment of delinquent patients with schizophrenia at the interface of health and justice system is described in a case report from swiss Forensic services. Authors point out that prison environments are difficult for individuals who lack social competences as a consequence of a severe mental disorder (Steinau et al.).

In general research in correctional institutions offers the option for a better understanding of clinical conditions which are rare in the community but common in prison. Evidence about individuals suffering from ASPD or of individuals who self-harm or act violently is necessary for improving treatment and prevention strategies in those cases. Although prison mental health care is often seen as strictly separated from community health provision this is in fact not true. In many cases prison psychiatry offers treatment for mentally disturbed offenders that from an early point have got lost in community health care. After mentally ill individuals are released from prison successful integration in community mental health care can help to prevent reoffending. Research collaboration of prison mental health professionals with providers of mental health in the community offers excellent opportunities for better understanding of factors that hinder the reintegration process. The editors are convinced that all clinicians working inside correctional institutions should be strongly encouraged to get involved in clinical research helping to develop evidence-based treatment strategies for the specific challenges of psychiatric work in prison.

## Author Contributions

AO-W wrote the first draft of the manuscript. NK und BV provided critical revison of the manuscript and important intellectual contributions. AO-W, NK, and BV read and approved the submitted version.

### Conflict of Interest

The authors declare that the research was conducted in the absence of any commercial or financial relationships that could be construed as a potential conflict of interest.

